# The effect of rapid maxillary expansion in children: a meta-analysis^☆^^☆☆^

**DOI:** 10.1016/j.bjorl.2020.12.017

**Published:** 2021-02-16

**Authors:** Denise M.C. Santana, Vania S. Nogueira, Silvana A.M. Lima, Luciana P.A. Fernandes, Silke A.T. Weber

**Affiliations:** a Universidade Estadual Paulista (UNESP), Faculdade de Medicina de Botucatu, Departamento de Oftalmologia, Otorrinolaringologia e Cirurgia de Cabeça e Pescoço, Programa de Cirurgia e Medicina Translacional, Botucatu, SP, Brazil; b Universidade Estadual Paulista (UNESP), Faculdade de Medicina de Botucatu, Botucatu, SP, Brazil; c Universidade Estadual Paulista (UNESP), Faculdade de Medicina de Botucatu, Departamento de Enfermagem, Botucatu, SP, Brazil; d Universidade Estadual Paulista (UNESP), Faculdade de Medicina de Botucatu, Departamento de Dermatologia e Radioterapia, Botucatu, SP Brazil; e Hospital Estadual de Botucatu, Laboratório do Sono do Hospital das Clínicas, Botucatu, SP Brazil; f Universidade Estadual Paulista (UNESP), Faculdade de Medicina de Botucatu, Departamento de Oftalmologia, Otorrinolaringologia e Cirurgia de Cabeça e Pescoço, Botucatu, SP, Brazil

**Keywords:** Children, Rapid maxillary expansion, Transverse maxillary deficiency, Mouth breather, Meta-analyses

## Abstract

**Introduction:**

Craniofacial growth is modified by chronic mouth breathing. Rapid maxillary expansion leads to separation of the mid-palatal suture, improving the occlusion and the upper airway size.

**Aim:**

Systematically evaluate scientific articles on the effects of rapid maxillary expansion on airway dimensions and classify the quality of the evidence of the information.

**Methods:**

Searches on PUBMED, LILACS, EMBASE, SCOPUS, WEB OF SCIENCE and COCHRANE, as well as in the grey literature were performed. The articles found were selected and evaluated both for the risk of bias (ROBINS-I) and for the quality of evidence (GRADE).

**Results:**

Of the 309 works found, 26 papers were selected for full reading, of which 22 were excluded. Data compilation and analysis were performed in four papers, two being controlled non-randomized clinical trials and two non-randomized and uncontrolled clinical trials. No randomized clinical trial was found.

**Conclusions:**

The meta-analysis found an increase in the internasal and inter-zygomatic distances and oropharyngeal volume after rapid maxillary expansion, which, together with clinical findings, makes the recommendation favorable to the intervention. The quality of the evidence for each outcome was considered very low.

## Introduction

The influence of breathing on the development of craniofacial structures has been widely discussed in the literature.^1^ According to Moss’ Functional Matrix hypothesis,^2^ nasal breathing leads to adequate growth and development of the craniofacial complex, together with other functions, such as chewing and swallowing.

Many studies have shown an association between respiratory disorders, craniofacial changes, and dental malocclusion.^3^, ^4^, ^5^, ^6^ Mouth breathing during the growth phase is considered an important factor, which is responsible for the sequence of events that commonly results in changes in growth, cranial and maxillomandibular development, resulting from predisposition due to craniofacial morphology; or it may be caused by several alterations, such as nasal septum deviation with obstruction, allergic rhinitis, nasal turbinate hypertrophy and hypertrophy of palatine and pharyngeal tonsils.^5^, ^6^ In mouth breathers, correlation has been reported between the upper airway anatomical conformation and changes in craniofacial morphology.^6^, ^7^ The frequently associated anatomical features are: maxillary atresia, retracted position of the maxilla and mandible, narrowing of the oropharynx and vertical growth pattern, as well as posterior open and posterior crossbite with hypotonic lips and atypical swallowing patterns.^5^, ^8^ The oropharynx is the middle part of the pharynx, located between the soft palate and the upper border of the epiglottis; it is the posterior part of the oral cavity.

Changes in the middle third of the face, as well as myofacial changes and tongue position are reported as important factors for the persistence of obstructive respiratory disorders in children after adenotonsillectomy, or as a cause in adolescents and young adults.^6^, ^9^

The correction of this craniofacial alteration is done through rapid maxillary expansion (RME), which has been reported to be an effective treatment for residual obstructive sleep apnea (OSAS) in children.^10^ RME is achieved through an orthodontic appliance with a laterally progressive opening mechanism using a screw that is activated daily. The orthopedic movement is achieved by opening the palatal suture, which remains open until approximately the age of 12, or is newly calcified in adolescence.^11^ According to Baratieri et al.,^12^ RME could not only produce dento-alveolar changes in cross-sectional correction, but it would also have implications for the nasal complex, as described by Lagravere et al.^13^ and Baratieri et al.^12^

Based on these favorable results, the quality of the evidence favoring the RME intervention and its degree of recommendation as a corrective treatment of the upper airway dimensions were questioned, with persistent results in the medium and long term.

Thus, it was proposed to conduct a systematic review of the effect of rapid maxillary expansion on the airway of young children and adolescents, with an analysis of the quality of the evidence and the degree of recommendation favoring the intervention.

The aim of this systematic review was to evaluate the therapeutic efficacy of RME in mouth breathing children and long-term respiratory disorders, regarding the improvement in breathing pattern, in addition to the objective anatomical craniofacial measurements, before and after the intervention, at 3 months, and at follow-up of at least 12 months, as well as to evaluate the quality of the evidence in the scientific literature and the degree of recommendation.

## Methods

The systematic review was carried out according to the recommendations of PRISMA (www.prisma-statement.org.), with a protocol registered on PROSPERO (registration number CRD42019119650).

The PICO strategy used in this systematic review is described in Table 1.

**Table 1:** Framework for elaborating the question, PICO.

Population	Pre-adolescents (mean age 12 years, maximum age 14 years) with obstructive respiratory disorders
Intervention	Rapid maxillary expansion
Comparative group	Without rapid maxillary expansion intervention
Outcomes	Restoration of the normal breathing pattern, analyzed clinically, by report and/or by measurements.
	1. Cephalometric radiographic exams, which are: Intermolar distance; Intercanine distance; Internasal distance; and interzygomatic distance
	2. Volumes: Nasal volume and pharyngeal volume
	3. Clinical report of mouth, nasal or mixed breathing before and after RME.

Prospective controlled intervention studies, with and without randomization, and case series studies were included. The target population for the intervention included children up to 14 years of age, presenting with mouth breathing and/or obstructive respiratory disorders, who underwent rapid maxillary expansion, without surgical assistance, while excluding studies involving adolescents over 14 years and 11 months of age, adults or patients with craniofacial and/or neurological genetic syndromes.

Two reviewers (SATW and DMCS) independently selected the studies identified by the literature search. Disagreements were solved by consensus.

The following characteristics were extracted from the studies: design, allocation methods, randomization, blindness, number, sex, and age of participants, followup after intervention, imaging test (CT or X-ray), craniofacial measures (intermaxillary distance, nasal or oropharyngeal volumes).

Each study was analyzed by the Robins-I tool, Cochrane, to assess the risk of bias in non-randomized studies of interventions.

The Review Manager 5.3 (RevMan) program was used to combine data from the same measurements from the included studies in a meta-analysis.^14^

The quality of the evidence of the effect size of each outcome was analyzed by the GRADE instrument, with a classification of high, moderate, low or very low.

This paper was exempted from obtaining an ethical opinion by the Human Research Ethics Committee of the Universidade Estadual Paulista Júlio de Mesquita Filho according to letter 136/2017-CEP, as it is a systematic literature review.

## Results

**Figure 1:**
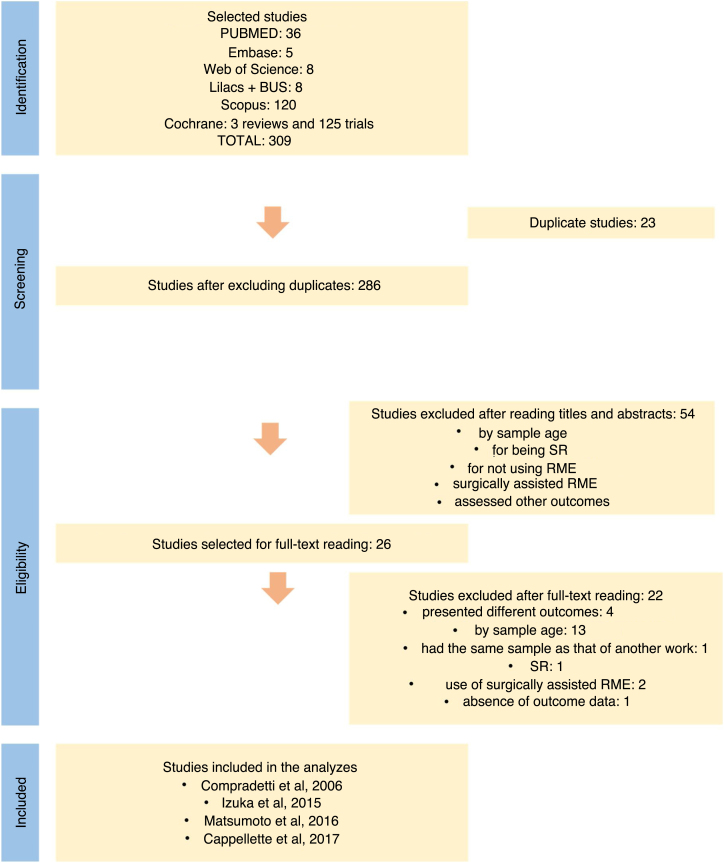
Flowchart of systematic review. Source: Adapted from PRISMA.

Of the 309 studies identified from searches in the electronic databases, 26 articles were evaluated in full, of which four remained for the final analysis, two of which were controlled non-randomized clinical studies,^15^, ^16^ and two non-randomized and uncontrolled clinical studies.^17^, ^18^ No randomized clinical trial was found (Fig. 1).

The detailed characteristics of the included studies are shown in Table 2. The detailed intervention effects of each included study are shown in Table 3.

**Table 2:** Characteristics of the included tests.

Study	Compadretti et al., 2006	Cappellette Jr. et al., 2017	Izuka et al., 2015	Matsumoto et al., 2016
Design	Non-randomized controlled clinical trial	Non-randomized controlled clinical trial	Non-randomized uncontrolled clinical trial	Non-randomized uncontrolled clinical trial
Patients	27 children: 13 boys, 14 girls	23 children: 11 boys, 12 girls	25 children	29 children
Age	Mean of 5–13 years (SD = 9.5 years ± 2.1)	Mean of 9.6 years (SD = 2.3 years)	Mean of 10.5 years (6–13 years, SD = 2.2)	7–10 years
Previous features	Upper arch constriction	Upper arch constriction	Mouth breathing	Mouth breathing
	Absence of natural causes for nasal respiratory failure	Mouth breathing	Maxillary atresia	Mixed dentures
		Mixed dentures	Posterior crossbite	Unilateral or bilateral posterior crossbite
	Enlarged adenoids		Nasal obstruction	
	Obstructive septum			
Further tests	RMM	Quality of life questionnaire	Quality of life questionnaire	Acoustic Rhinometry
	AR	EOL: Rhinoscopy; Oroscopy; Nasal fibroendoscopy	EOL: Rhinoscopy; Oroscopy; Nasal fibroendoscopy	Computerized Rhinomanometry
	Audiometry			
	Tympanometry			Nasal fibroscopy
	PA X-rays: Internasal distance; Interzygomatic distance; Intermaxillary distance	Orthodontic examination: atresia of the upper arch; 3 min test with closed lips; Computed tomography (before RME and 3 months after RME)	Computed tomography	PA X-rays: NC-NC (corresponding to the internasal width); JL-JL (corresponding to maxillary width)
Group control	24 children: 16 boys, 8 girls	15 children: 9 boys, 6 girls	–	–
Age, control group	Mean of 8–12 years (10.2 years ± 1.5 SD)	10.5 years (SD = 1.9 years)	–	–
Previous characteristics, control group	Without obstruction (confirmed by nasopharyngoscopy)	Not reported	–	–
Tests, control group	RAM and AR (first visit and after 11 months on average)	Computed Tomography (before RME and 3 months after RME)	–	–
Intervention	RME with Hyrax	RME with Hyrax	RME with Bierderman	RME with Haas
Country	Italy	Brazil	Brazil	Brazil
Follow-up	12 months ± 0.8 SD (mean of 10.3–13.6 months)	3 months	3 months	30 months
Dolphin Imaging v.11.7 software CT + Reconstruction	–	Volumetric measurements and comparisons between the images of both groups were taken to Dolphin and evaluated in “airway volume” in 3 views: sagittal, coronal, and axial.	Used to assess the patients' upper airway, using the following measures: ANF: width of the anterior portion of the nasal floor; PNF: width of the posterior portion of the nasal floor; VNN: volume of the nasopharynx and nasal cavity	–
Conclusion	The improvement in nasal breathing after RME is due to the significant increase in nasal width.	RME showed a significant increase in the transverse dimension of the maxilla and a significant increase in nasal and oropharyngeal volumes.	Significant increase in transverse maxillary distances and significant increase in nasal and oropharyngeal volumes.	RME significantly increased the maxillary and nasal transverse distances.

**Table 3:** Effects of Interventions for trials.

Compadretti et al., 2006	Non-randomized controlled clinical trial		
Cephalometric measurements (mm)	T1 (3 months)	T2 (≥12 months)	Mean – SD
Nasal cavity width	Not reported	Not reported	2.2 ± 2.1
Intercanine distance	Not reported	Not reported	Not reported
Intermolar distance	Not reported	Not reported	4.8 ± 10.6
Interzygomatic distance	Not reported	Not reported	7.0 ± 8.6

Cappellette Jr, et al., 2017	Non-randomized controlled clinical trial			
	Control Group		Experimental Group	
Dolphin measurements (mm^3)^	T0 (pre-RME) Mean – SD	T1 (3 months) Mean – SD	T0 (pre-RME) Mean – SD	T1 (3 months) Mean – SD
Nasal volume	34,426.0 ± 5,059.0	34,488.7 ± 5,088.9	33,418.7 ± 6,107.6	38,450.6 ± 6,329.1
Oropharyngeal volume	7,531.0 ± 1,535.0	7,572.4 ± 1,526.4	10,262.3 ± 2,421.1	12,955.1 ± 2,942.8

Izuka et al., 2015	Non-randomized uncontrolled clinical trial	
Cephalometric measurements (mm)	T0 (pre-RME) Mean – SD	T1 (3 months) Mean – SD
Nasal cavity width	Not reported	Not reported
Intercanine distance	16.3 ± 1.7	19.1 ± 1.8
Intermolar distance	22.6 ± 2.5	25.4 ± 3.0
Interzygomatic distance	Not reported	Not reported
**Dolphin measurements (mm^3)^**	**T0 (pre-RME) Mean – SD**	**T1 (3 months) Mean – SD**
Nasal volume	6114.4 ± 3490.4	7760.5 ± 3841.4
Oropharyngeal volume	6378.2 ± 2357.5	7826.8 ± 4109.9

Matsumoto et al., 2016			
Cephalometric measurements (mm)	T0 (pre-RME) Mean – SD	T1 (3 months) Mean – SD	T2 (≥12 months) Mean – SD
Nasal cavity width	24.25 ± 2.08	25.69 ± 2.22	27.98 ± 2.68
Intercanine distance	Not reported	Not reported	Not reported
Intermolar distance	Not reported	Not reported	Not reported
Interzygomatic distance	58.88 ± 2.80	62.12 ± 2.70	66.29 ± 3.53

All studies were assessed for their risk of bias, using the ROBINS-I tool (Table 4), showing moderate to high risk of bias for “selection of the studied population”, “measurement of outcome”, and “selection of reported results”, being considered of moderate risk of bias, except that by Compadretti el al.,^15^ which was considered to have a risk of serious bias (Table 5).

**Table 4:** Assessment of risk of bias according to ROBINS-I tool.

ROBINS-I tool	Non-randomized controlled clinical trial		Non-randomized uncontrolled clinical trial	
	Compradetti et al., 2006	Cappellette Jr. et al., 2017	Izuka et al., 2015	Matsumoto et al., 2016
Confusion bias	High	Moderate	Low	Low
Selection bias	Moderate	Moderate	Moderate	Moderate
Classification bias	Low	Low	Low	Low
Bias due to deviation from intended intervention	Low	Low	Low	Low
Lack of data bias	High	Low	Low	Moderate
Outcome measurement bias	Moderate	Moderate	Moderate	Moderate
Bias in the selection of reported results	High	Moderate	Moderate	Moderate
Bias risk judgment	High risk of bias	Moderate risk of bias	Moderate risk of bias	Moderate risk of bias
	No risk of critical bias

**Table 5:** Summary of GRADE results.

Outcomes	Potential absolute effects * (95% CI)		Relative effect (95% CI)	No. of participants (studies)	Certainty of the evidence (GRADE)	Comments
	Risk with [comparison]	Risk with [intervention]				
Internasal distance	Average internasal distance was 0 (zero)	DM 1.78 higher (0.95 higher to 2.6 higher)	–	54 (2 observational studies)	⨁◯◯◯ Very low^a^, ^b^	
Oropharyngeal volume	Mean oropharyngeal volume was 0 (zero)	DM 2.18 higher (0.98 higher to 3.38 higher)	–	48 (2 observational studies)	⨁◯◯◯ Very low^a^, ^b^	
Interzygomatic distance	Interzygomatic mean distance was 0 (zero)	DM 2.75 lower (15.96 lower to 10.47 higher)	–	54 (2 observational studies)	⨁◯◯◯ Very low^a^, ^b^	
Nasal volume	Mean nasal volume was 0 (zero)	DM 3 higher (0.25 lower to 6.25 higher)	–	48 (2 observational studies)	⨁◯◯◯ Very low^a^, ^b^	

a High risk of selection bias.

b Small number of studies and patients.

Only the study by Compadretti et al.^15^ described the outcome “Restoration of nasal breathing” quantitatively, reporting that 42.8% of their sample changed from oral breathing to nasal breathing; the other authors reported only improvement in mouth breathing.

**Figure 2:**
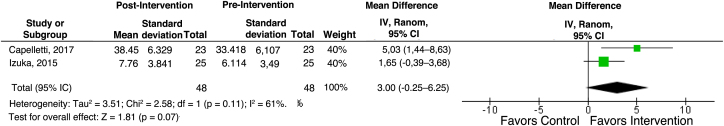
Meta-analysis of the nasal volume outcome, in mm^3^, 3 months after the intervention.

With a 3 month followup, the studies by Cappellette Jr. et al.,^16^ Izuka et al.^17^ and Matsumoto et al.^18^ reported the cephalometric data regarding the reassessment after the 3 month follow-up, but with different measurements, which made a meta-analysis impossible. As for the outcomes of volume measurements, the data obtained from the studies by Cappellette Jr. et al.^16^ and Izuka et al.^17^ enabled the meta-analysis. The nasal volume outcome was not significant to favor the intervention, with an average difference of 3.00 mm^3^ (95% CI -0.25–6.25) for a total of 48 patients (Fig. 2).

**Figure 3:**
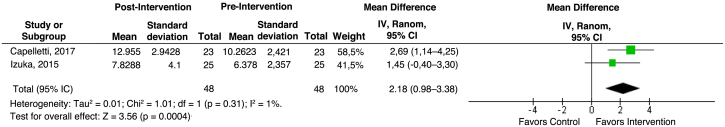
Meta-analysis of the oropharyngeal volume outcome, in mm^3^, after 3 months of intervention.

Regarding the oropharyngeal volume outcome, the meta-analysis favored the intervention, with a mean difference was 2.18 mm (95% CI -0.98–3.38) for a total of 48 patients (Fig. 3).

**Figure 4:**
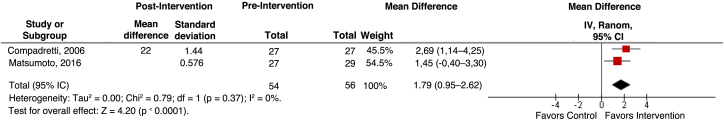
Meta-analysis of the internasal distance outcome, in mm, ≥ 12 months after the intervention.

**Figure 5:**
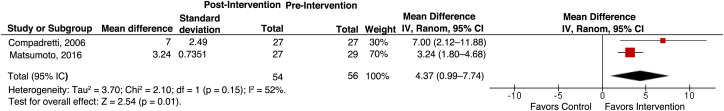
Meta-analysis of the outcome interzygomatic distance, in mm, ≥ 12 months after the intervention.

Only the studies by Compradetti et al.,^15^ and Matsumoto et al.^18^ performed a new evaluation of the maxillary opening outcome, after more than 12 months of follow-up, reporting the internasal and interzygomatic distance measurements, enabling a meta-analysis with favorable results from the intervention. The difference in mean internasal distance was 1.79 mm (95% CI 0.95–2.62), the interzygomatic distance was 4.37 mm (95% CI 0.99–7.74), including a total of 54 patients (Figure 4, Figure 5).

### Quality of evidence – GRADE

The grading of recommendations assessment, development and evaluation (GRADE) is a tool developed to assess the quality of evidence and the strength of recommendations. Table 5 shows the quality of evidence and its recommendation for each outcome assessed. Based on the studies included in this SR, the quality of the evidence favoring RME is very low for all outcomes.

## Discussion

Transverse maxillary deficiency associated with respiratory problems has been widely discussed by otolaryngologists and orthodontists due to the relationship between causes, effects, and treatments. In 1860 Angell^19^ introduced for the first time a technique that enabled the mid-palatal suture division, which he called maxillary expansion. The work drew the attention of the otolaryngologist Eysel, mentioned by Haas in 1961,^8^ who studied the effects of this maxillary expansion on the nasal cavity dimensions in 1886 and observed that, in the period after the maxillary expansion, several changes occurred in the maxilla, such as the increase in nasal width close to the mid-palatal suture.^6^

Since then, several studies have been performed following the same concept as RME therapy,^15^, ^16^, ^17^, ^18^ as an important treatment option for transverse maxillary deficiency, a form of maxillary hypoplasia reported as a risk factor for obstructive sleep disorders in children, adolescents and adults, and as the most important cofactor related to adenotonsillectomy therapeutic failure in children/adolescents with OSAS.^6^

The high interest in the subject was reflected in the number of articles found in the literature review, with more than 80% of the articles being published in the last 15 years. However, despite the RME results indicating an increase in the nasal structure and upper airway, few studies have focused on the pattern of breathing (if by nose or mouth) before and after treatment, with most studies mixing the population of children and adults and having a combination of surgically-assisted RME technique, which reduced the number of articles with the outcomes of interest to 26.

RME treatment has not clearly defined an age limit for its indication and best effectiveness. However, the ossification process in the mid-palatal suture during puberty hinders the expansion process, so that in adults an expansion with the opening of the suture is only achieved through an associated surgical process (surgically assisted RME).

In 2004, Ennes^20^ evaluated the degree of mid-palatal suture ossification in human skulls of different age groups, and saw that the mid-palatal suture ossification presents few points of radiolucency at the early adult stage, with space between the bony margins of the mid-palatal suture identified in the anterior segment and beginning of the middle, while in the group of children, no ossification bridges were identified. In this study, the age of 14 years revealed a retention of some space between the bony margins in the anterior and middle palatal segments of the mid-palatal suture, but already with more intense radiopacity. Thus, in adolescents over 14 years of age, RME would not achieve ideal results. Based on this data, for this review, studies that evaluated the effect of RME on the population of children and young adolescents up to 14 years were considered.

To our surprise, 13 of the 22 studies included a pediatric population up to 18 years old, some of these studies even performing RME and RME surgically assisted, and therefore they were excluded. Even in adolescents aged 12–14, there was no classification of the stage of puberty by validated protocols, such as Tanner's.^21^ The female population reaches puberty earlier, but there was also no difference in the analysis of results by gender in the studies, limiting their interpretation. As noted, age at the time of RME is an important factor in the effect of maxillary opening,^22^ so each age has its own response pattern. In 3 of the 4 included studies (Compadretti et al.,^15^ Cappellette Jr. et al.,^16^ Izuka et al.^17^), a wide variation in age was found with an interval of 7–8 years between the younger and the older. Only the study by Matsumoto et al.,^18^ delimited the population, narrowing the age group variation by 3 years, with patients between 7 and 10 years old. Followups such as the rhinopharynx and hypopharynx were not specified. These facts were considered to have a moderate selection bias, contributing to reduce the quality of the evidence.

Regarding quality of life questionnaires, no study used a more objective assessment of nasal/mouth breathing by a questionnaire validated in Brazil, such as the SNOT-22,^23^ which is a useful tool in assessing the improvement of pre- and post-intervention symptoms.

As the measurements performed were presented in absolute values and not related to the body surface or other measurement of the skull standardized for age and gender, there is a large numerical variation, not allowing to state which target population would obtain the greatest effect of the intervention, knowing that the gain in opening the maxilla or in volumes of the nasal and pharyngeal cavities is not linear for age and depends on gender. This great variation in the measurements is observed in the high values of the standard deviation in the studies.

It is also important to note that no study makes reference to normal, and therefore there are no reports in the studies of how many children were below the measures considered normal before the intervention and how many were within the normal range after the intervention, making it difficult to interpret the magnitude of the effect.

Regarding diagnosis and treatment planning, data from craniocephalometry and/or computed tomography have been used in the analysis of the effectiveness of treatment with RME. The measurements evaluated as important outcomes are those representing the measurements of the transverse opening of the maxilla, such as the internasal, inter-canine, intermolar, and inter-zygomatic distances and the cephalometric angles, which correspond to the improvement in the projection of the maxilla and mandible in relation to the skull base. It is worth mentioning that Izuka et al.^17^ reported as an internasal distance the measurement of the transverse width of the anterior portion of the nasal floor, and accessed after the demarcation of 2 points to the right and left of the nasal ridges, in the canine region. The other studies did not make the limits so clear, generating bias of no definition and, therefore, not guaranteeing the reproducibility of the study.

3D reconstructions of nasal and oropharyngeal volumes by the Dolphin program of tomographic images are also measurements that allow the evaluation of the expansion of craniofacial structures.^15^, ^16^ However, there was no uniformity in the measurements reported in the analysis in each study, making it impossible to meta-analyze all reported data, except for internasal and interzygomatic distances, as well as nasal and oropharyngeal volumes, grouping only 2 studies for each measurement, which reduced the weight of the intervention effect.

Evaluation 3 months after active maxillary expansion is part of the RME protocol, being considered the end of the expansion stabilization period. Although 3 of the 4 studies reported data from this reevaluation time, only 2 studies used equal measurements, allowing for meta-analysis, but with less weight for the evidence of the intervention due to the small number of children involved and the bias already mentioned.

The longest follow-up was reported by two studies, Compradetti et al.,^15^ and Matsumoto et al.,^18^ with effects favoring the intervention, but with the same restrictions on the quality of the evidence as mentioned above.

Although randomization for RME intervention requires a complex methodological design to simulate an expander device with daily sham screw rotations, new studies with better methodological quality and standardized craniocephalometric measurements should be planned. Because children are considered to be a vulnerable population, the design must guarantee ethical support.

The evaluation of the quality/level of evidence of RME favoring the increase in craniofacial dimensions resulted in “very low”, with all studies showing moderate to severe selection bias in the measurement of outcomes, in the selection of reported results, and in the risk of bias. Despite the low quality, meta-analyzes for internasal distance, inter-zygomatic distance, and oropharyngeal volume showed an effect favoring the intervention, with heterogeneity considered low. This positive effect is also seen in the symptoms. Thus, although it is not possible to identify a group that would benefit the most and there is not yet a good level of evidence, there is a weak recommendation favoring RME for the management of maxillary deficiency.

Limitations of the included studies were evidenced regarding the absence of individualized data, risk of bias, differences in the designs of the tested devices, variations in the follow-up time, wide age range, lack of standardization of the “normal”, in addition to the lack of standardization in the tested measurements. However, the results showed low heterogeneity favoring the intervention, in addition to the clinical results also being favorable. Thus, there is a weak recommendation favoring the intervention.

## Conclusion

This systematic review shows that RME results in an increase in the transverse dimensions of the maxilla and upper airway volumes after 3 months and with a followup greater than 12 months. The quality of evidence for these outcomes is considered to be very low.

## Conflicts of interest

The authors declare no conflicts of interest.
